# The *Aquilegia pubiflora* (Himalayan columbine) mediated synthesis of nanoceria for diverse biomedical applications

**DOI:** 10.1039/d0ra01971b

**Published:** 2020-05-20

**Authors:** Hasnain Jan, Muhammad Aslam Khan, Hazrat Usman, Muzamil Shah, Rotaba Ansir, Shah Faisal, Niamat Ullah, Lubna Rahman

**Affiliations:** Department of Biotechnology, Quaid-i-Azam University Islamabad 45320 Pakistan hasnainjan@bs.qau.edu.pk; Department of Biotechnology, International Islamic University Islamabad Pakistan muhammadaslamkhanmarwat@gmail.com; Department of Chemistry, Quaid-i-Azam University 45320 Islamabad Pakistan; Department of Biotechnology, Bacha Khan University Charsadda KP Pakistan

## Abstract

Herein, we report an eco-friendly, facile, one-pot, green synthesis of nanoceria for multiple biomedical applications. In the study, cerium oxide nanoparticles (CeO_2_-NPs) were synthesized using a simple aqueous extract of *Aquilegia pubiflora* as an effective reducing and capping agent. The biosynthesized nanoparticles were characterized *via* UV-vis spectroscopy, X-ray powder diffraction (XRD), high-performance liquid chromatography (HPLC), Fourier transform infrared spectroscopy (FTIR), scanning electron microscopy (SEM), transmission electron microscopy (TEM), and Raman spectroscopy. The NPs were highly stable, exhibited high purity, and had a spherical morphology and mean size of 28 nm. FTIR and HPLC studies confirmed the successful capping of bioactive compounds on the nanoparticles. The well-characterized NPs were evaluated for a number of biomedical applications, and their antimicrobial (antifungal, antibacterial, and antileishmanial), protein kinase inhibition, anticancer, antioxidant, anti-diabetic and biocompatibility properties were studied. Our results showed that the *Aquilegia pubiflora* mediated CeO_2_-NPs were highly active against fungal strains, compared to the tested bacterial strains, with *Aspergillus niger* resulting in the largest zone of inhibition (15.1 ± 0.27 mm). The particles also exhibited dose dependent leishmanicidal activity with significant LC_50_ values toward both the amastigote (114 μg mL^−1^) and promastigote (97 μg mL^−1^) forms of the parasite *Leishmania tropica* (KWH23). The NPs were found to be moderately active against the HepG2 cell line, showing 26.78% ± 1.16% inhibition at 200 μg mL^−1^. Last but not least, their highly biocompatible nature was observed with respect to freshly isolated human red blood cells (hRBCs), making the greenly synthesized CeO_2_-NPs a novel candidates for multidimensional medical applications.

## Abstract 2

Graphical illustration of eco-friendly, facile, one-pot, green synthesis of nanoceria for multiple biomedical applications.

## Graphical abstract

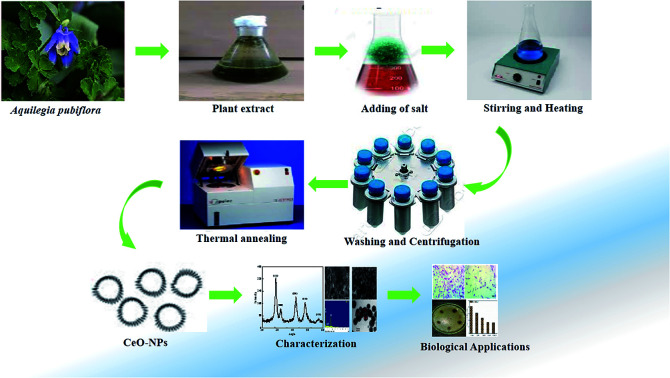

## 1. Introduction

The availability of cerium oxide (CeO_2_), one of the most abundant rare earth oxides, has encouraged tremendous research into the development of functional cerium nanoparticles for diverse applications.^1^ The unique properties of CeO_2_ have been utilized in bio-sensing,^2^ ultraviolet absorbance,^3^ catalysis, electrochemical cells, corrosion prevention, glass polishing, and thermal coatings.^4^

In recent years, CeO_2_-NPs have also received much attention from the scientific community in relation to medicine.^1^ Owing to their excellent radical scavenging abilities, different studies have demonstrated that CeO_2_-NPs can alleviate the symptoms of many oxidative stress-related diseases, such as retinitis, chronic inflammation, neurodegeneration, diabetes, and cancer. However, the eco-friendly synthesis of high quality CeO_2_ is still a challenging task.^5^

Traditional chemical and physical methods are mostly utilized for the synthesis of CeO_2_-NPs. These include solution precipitation, hydrothermal,^6^ solvothermal,^7^ ball milling and thermal decomposition,^7^ spray pyrolysis,^8^ thermal hydrolysis,^9^ and sol–gel^10^ methods. However, such methodologies are time-consuming, require large amounts of energy, and produce hazardous chemicals, making them environmentally unfriendly. Recently, bio-assisted synthesis methods that use living organisms as capping and stabilizing agents have been proven to be efficient, inexpensive, and safer routes for the preparation of cerium oxide nanoparticles.^11^

The green synthesis of CeO_2_-NPs using plant extracts has already been demonstrated, however, limited data is available regarding their diverse biological applications, including their antimicrobial, antileishmanial, antioxidant, anti-diabetic, and anti-cancer potential. Here, we report the rapid, room temperature synthesis of CeO_2_-NPs *via* a completely eco-friendly procedure, which involves the use of a simple aqueous extract of *Aquilegia pubiflora* as an effective oxidizing/reducing and capping agent. The medicinal uses of *A. pubiflora* are well documented, and it is commonly used in hepatitis and skin burn treatment, wound healing, and the treatment of jaundice, and gynecological, circulatory and cardiovascular problems^12–15^.

To the best of our knowledge, this is the first ever report on the *Aquilegia pubiflora* mediated synthesis of CeO_2_-NPs. The synthesized NPs were characterized *via* UV-vis spectroscopy, Fourier transform infrared spectroscopy (FTIR), X-ray diffraction (XRD), scanning electron microscopy (SEM), transmission electron microscopy (TEM), energy dispersive X-ray (EDX) analysis, and Raman spectroscopy. The well-characterized NPs were then investigated for their anticancer, antimicrobial, anti-diabetic, and antioxidant potential. Moreover, to confirm their bio-safe nature, the NPs were evaluated for biocompatibility against freshly isolated human red blood cells (hRBCs).

## 2. Materials and methods

### 2.1. Collection and processing of the plant material

The herbs used in the current study were collected from Swat District (Miandam), Khyber Pakhtunkhwa, Pakistan. The plant was taxonomically identified as *Aquilegia pubiflora* at the Department of Botany, Bacha Khan University, Charsadda, and later verified at the Department of Plant Sciences, Quaid-i-Azam University Islamabad, Pakistan.

The fresh and fine leaves of the plant were excised into small pieces using a sterile surgical blade, rinsed well with distilled water to remove any dust particles and impurities, followed by drying in the shade. The well-dried leaves were then ground into a fine powder in a Wiley mill and stored at 25 °C for aqueous extraction.

Aqueous leaf extract was prepared by adding 30 g of fine powder to a flask (500 mL) containing 200 mL of distilled water, which was sonicated continuously for 10 min and kept in a shaking incubator at 200 rpm for two days at 37 °C. The prepared extract was initially filtered twice using nylon cloth to remove solid residue. The obtained extract was further filtered three times using Whatman filter paper No. 1 to remove any remaining particulates. The fresh filtrate obtained was then processed for further use.

### 2.2. Biosynthesis of the CeO_2_ nanoparticles

Briefly, 3 g of cerium(iii) chloride heptahydrate (CeCl_3_·7H_2_O; Sigma-Aldrich) was added to 100 mL of extract and kept under magnetic stirring at 60 °C for 2 h. Once the reaction was completed, the mixture was allowed to cool down at 25 °C, followed by centrifugation at 10 000 rpm for 10 min. The supernatant was discarded, and the remaining pellet was washed thrice with distilled water, poured into a clean Petri plate and oven dried at 90 °C. The dried material was then ground into fine powder in a pestle and mortar and calcinated for 2 h at 500 °C to remove any impurities. The annealed powder was stored in an air-tight glass vial, labelled as CeO_2_-NPs, and was further used for physical characterization and biological applications.

### 2.3. Physicochemical and morphological characterization

Several characterization techniques, including UV spectroscopy, Fourier transform infrared spectroscopy (FTIR), high-performance liquid chromatography (HPLC), X-ray diffraction (XRD), scanning electron microscopy (SEM), transmission electron microscopy (TEM), energy dispersive X-ray (EDX) analysis, and Raman spectroscopy were used to evaluate the structural, chemical, vibrational and morphological properties of the biosynthesized CeO_2_-NPs. To determine the phase and crystalline nature, XRD (Model-D8 Advance, Germany) studies were carried out in the 2 theta range of 10°–55°. Diffraction data was obtained using Cu Kα radiation (wavelength: 1.5406 Å) *via* analyzing 1 mg of powdered CeO_2_-NPs, with a step-time of 0.55 seconds and a scanning step size of 0.03° s^−1^. To calculate the crystallite size, the Scherrer equation was used as follows:*D* = *Kλ*/*β* cos *θ*where *D* denotes the crystallite size, *k* represents the shape factor (0.94), *λ* is the X-ray wavelength, which was 1.5421 Å, and *β* and *θ* refer to the full-width-at-half-maximum in radians and the Bragg angle, respectively. In order to determine the associated functional groups on the NPs as a result of capping and reducing agents within the extract, FTIR spectra were obtained in the spectral range of 400 cm^−1^ to 4000 cm^−1^ and HPLC studies were carried out. Morphological properties were examined through scanning electron microscopy (SEM) and transmission electron microscopy (TEM), while the elemental compositions of samples were determined using energy dispersive X-ray spectroscopy (EDS). Raman spectra of the biogenic CeO_2_-NPs were obtained in the spectral range of 200 cm^−1^ to 700 cm^−1^ to find the vibrational modes of the NPs.

Size distribution and zeta potential (*ζ*) data were obtained using Zetasizer Nano-ZS apparatus (Malvern Instruments, Worcestershire, UK). Briefly, a tenfold dilution of sample (1 mL) in ultra-pure water (1 mL) was added to a particle size analyzer at room temperature. Zeta potential and size distribution data were recorded using inbuilt software based on the Helmholtz–Smoluchowski equation.^16^

### 2.4. Anti-bacterial assays

The agar well diffusion method, as documented previously, was used with some modifications for the assessment of the *in vitro* bactericidal potential of the CeO_2_-NPs against different pathogenic strains.^17^ Briefly, microbial cultures were grown overnight in a sterilized nutrient broth medium at 37 °C in a shaking incubator. The turbidity of the culture was standardized to an OD of 0.5, corresponding to MacFarland standards (with a seeding density of 1 × 10^8^ CFU mL^−1^). From the standardized microbial cultures, 50 μL of inoculum was dispensed in Petri plates with solid agar medium and spread uniformly using sterile cotton swabs. A 5 mm borer was used to make wells. Each well was carefully filled with 10 μL of test sample. Accordingly, the seeded plates were also labelled. Cefixime and roxithromycin (standard antibiotics) and DMSO acted as positive and negative controls, respectively. After incubation for 24 h at 37 °C, each well loaded with a test sample was observed for the appearance of a zone of inhibition (ZOI). The diameter of the zone was measured in mm with a Vernier caliper. All experiments were conducted in triplicate and were statistically analyzed using Statistix 8.1. One-way ANOVA was used to check the significant mean difference with Tukey's HSD for *post hoc* analysis. *P* < 0.05 was used to define a significant result. All graphs were made using Origin 8.1.

### 2.5. Anti-fungal assays

The agar well diffusion method was used to evaluate the antifungal potency of the CeO_2_-NPs.^18^ In brief, sterile SDA (Sabouraud dextrose agar) medium was poured into autoclaved plates and 100 μL spore suspensions of every fungal strain were taken and swabbed onto the solidified media in the Petri plates. Each sample to be tested (10 μL) was poured into the wells, and the plates were also labeled accordingly. DMSO and clotrimazole were used as negative and positive controls, respectively. Plates were incubated at 28 °C for 24–48 h to clearly visualize the fungal growth. After incubation, each well loaded with test samples or controls was checked for the appearance of a ZOI (zone of inhibition). The diameter of the ZOI was measured using a Vernier caliper to the nearest mm. All experiments were conducted in triplicate and were statistically analyzed using Statistix 8.1. One-way ANOVA was used to check the significant mean difference with Tukey's HSD for *post hoc* analysis. *P* < 0.05 was used to define a significant result. All graphs were made using Origin 8.1.

### 2.6. Anti-leishmanial assays (promastigotes and amastigotes)

The anti-leishmanial potential of the biogenic CeO_2_-NPs was investigated based on the MTT cytotoxic activities against amastigote and promastigote cultures of *L. tropica* KWH23 (Department of Biotechnology, BKUC, Pakistan).^19^ M199 media with 10% fetal bovine serum was used to culture leishmanial parasites (culture density: 1 × 10^6^ cells per mL). The activity was examined in a 96-well plate at concentrations ranging from 200–25 μg mL^−1^. DMSO was used as a blank and amphotericin B was used as a positive control. The seeded 96-well plate was incubated at room temperature for 72 h. Optical density (OD) measurements were carried out at 540 nm, while all living cultures were counted using an inverted microscope. Table curve software was used to calculate the respective LC_50_ values. Percentage inhibition values were calculated using the following formula:
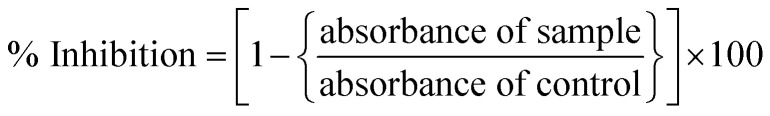


### 2.7. Protein kinase assays

Protein kinase enzyme inhibition assays were performed to verify the protein kinase inhibition abilities of the biogenic CeO_2_-NPs with slight modifications.^18^ The medium (TSB broth) was prepared, autoclaved at 121 °C for 20 minutes, and incubated for 24 h to detect any impurities. Both *Streptomyces* strains and autoclaved TSB broth were kept in a shaking incubator at 30 °C for 24 h. Inorganic salt starch agar (ISP4) minimal medium was used for the growth of fungi. Except for agar, starch and calcium carbonate, the remaining ingredients were added with 300 mL of distilled water and mixed constantly with a stirrer. Starch, calcium carbonate and agar were dissolved in 200 mL of distilled water in a separate flask. Both solutions were finally mixed together to make up the final concentration in 500 mL of medium. The medium was autoclaved and poured into autoclaved Petri plates under antiseptic conditions to avoid contamination. The media was kept to allow it to solidify, and *Streptomyces* strains in culture broth were uniformly spread on the surface of the medium with autoclaved cotton-buds. A sterile borer was used for making wells, and molten agarose was added to the bottoms of the wells to avoid the diffusion of the test samples. Afterwards, 20 μL of serially diluted sample was added to each well and it was kept intact for a few minutes to avoid the aggregation of poured sample in the well. Surfactin and DMSO were used as positive and negative controls, respectively. Petri plates were properly labeled and kept for incubation at 37 °C for 24–48 h. Using a Vernier caliper, zones of inhibition were measured in mm after 24 h and 48 h, respectively.

### 2.8. Cytotoxicity against the HepG2 cell line

The standard protocol from ref. 20 with slight modifications was used to assess the anti-proliferative potential of the CeO_2_-NPs against human hepatocellular carcinoma cells (ATCC HB-8065). HepG2 cells were cultured (37 °C in a humidified 5% CO_2_ atmosphere) in DMEM containing 10% fetal calf serum (FCS), supplemented with 100 U mL^−1^ penicillin, 100 μg mL^−1^ streptomycin, 2 mM l-glutamine, and 1 mM Na-pyruvate. 0.5 mM trypsin/EDTA was used for cell harvesting at room temperature. Nanoparticles were suspended in deionized water and sonicated for 30 min prior to experiments. HepG2 cells (90% confluency) were seeded in a 96-well plate at a density of 12 000 cells per well and allowed to adhere for 24 h at 37 °C. Subsequently, cells were treated with samples at 100 μg mL^−1^ for 24 h. 50% pre-chilled trichloroacetic acid (TCA) was then used for cell fixation, followed by incubation at 4 °C for 1 h; later, samples were rinsed with deionized water three times. The plate was then air-dried and the cells were stained with SRB dye (0.05%), followed by room-temperature incubation (30 min) and washing with acetic acid (1%) for removing unbound dye. Non-treated cells (NTC) and the drug doxorubicin (30 μM) were used as controls. Images were taken using a light microscope (Olympus CK2) equipped with a digital camera. A microplate reader (Platos R 496, AMP) was used to obtain abs values at 565 nm. Percentage viability values relative to an untreated sample were calculated using the formula:
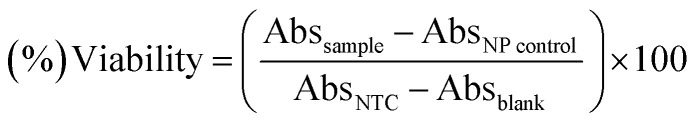
where Abs_sample_ and Abs_NTC_ denote treated samples and non-treated cells, respectively. Abs_NP control_ and Abs_blank_ represent the background OD values measured from NP and media samples alone, respectively. The assay was repeated twice with triplicates measurements of each sample.

### 2.9. Anti-diabetic assays

α-Amylase and α-glucosidase inhibition bioassays were performed to investigate the anti-diabetic potentials of samples.

#### 2.9.1. α-Amylase inhibition assay

A previously reported chromogenic method^21^ was used to evaluate the potential of the NPs to inhibit α-amylase (Sigma-Aldrich). The enzyme (1 U mL^−1^) was prepared in phosphate buffer (0.1 M; pH 6.8) and was thoroughly mixed with 4-nitrophenyl-α-d-maltopentaoside (5 mM) solution. An aliquot of sample was then added to the reaction mixture, followed by incubation for 30 minutes at 37 °C. The reaction was quenched by the addition of the same volume of sodium carbonate (1 M). The absorbance values of samples were recorded using a microplate reader (405 nm), and the activity was measured as % inhibition as follows:
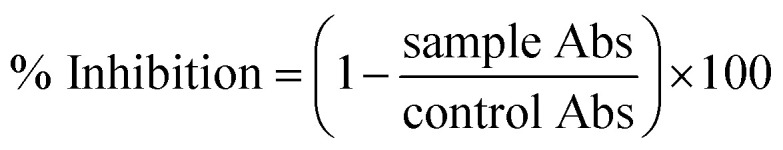


#### 2.9.2. α-Glucosidase inhibition assay

To further asses the antidiabetic potential, α-glucosidase inhibition assays were also performed *via* a chromogenic method,^21^ using a polyethylene filter (0.45 μm) end-capped column. In brief, an aliquot of test sample was added to a reaction mixture containing intestinal fluid (1 mL) consisting of 4-nitrophenyl-α-d-glucopyranoside (5 mM; 4NPG; Sigma) and incubated at 37 °C. The reaction was terminated after 30 min of incubation by adding sodium carbonate (1 M) solution. The absorbance of the tested sample was recorded at 405 nm and the activity was measured as a percentage inhibition value using the formula:
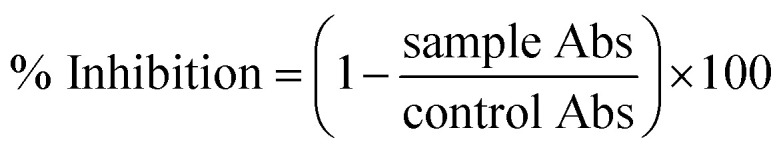


### 2.10. Antioxidant assays

#### 2.10.1. Free radical scavenging assay (FRSA)

2,2-Diphenyl-1-picrylhydrazyl (DPPH) free radicals were used for estimating the antioxidant activity of the CeO_2_-NPs. 200, 100, 50, 25, and 12.5 μg mL^−1^ concentrations were used in the reaction mixture, and the assays were repeated in triplicate. In brief, 180 μL of DPPH solution (4.80 mg/50 mL of methanol) was added to 20 μL of test sample at each concentration in a 96-well plate, which was incubated for 30 minutes at 37 °C. Ascorbic acid was used as a positive control and DMSO was employed as a negative control. The absorbance of the reaction mixture was measured at 515 nm using a microplate reader, and the free radical scavenging potential was measured as a percentage using the following equation:
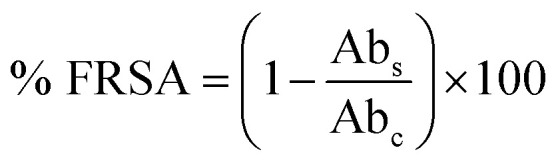
where Ab_c_ and Ab_s_ indicate the absorbances of the negative control and sample, respectively.

#### 2.10.2. Total antioxidant capacity (TAC)

The total antioxidant capacity (TAC) values of the CeO_2_-NPs were determined using phosphomolybdenum-based assays.^22^ The assay was performed at different concentrations, *i.e.*, 12.5 μg mL^−1^, 25 μg mL^−1^, 50 μg mL^−1^, 100 μg mL^−1^, and 200 μg mL^−1^. Phosphomolybdenum reagent was prepared by adding together 1.63 mL (0.6 M) of H_2_SO_4_, 1.6795 g (28 mM) of NaH_2_PO_4_, and 0.0247 g (4 mM) of ammonium molybdate. A reaction mixture of 100 μL of test sample (4 mg mL^−1^ DMSO) and 900 μL of reagent was allowed to incubate at 95 °C for 0.5 h in a water bath. After cooling, 200 μL of each sample was poured into separate wells in a 96-well plate. The absorbance values of the samples were taken at 630 nm using a microplate reader. The assays were executed in triplicate. Antioxidant activity values were expressed as micrograms of ascorbic acid equivalent per milligram (AAE μg mg^−1^) of the CeO_2_-NPs.

#### 2.10.3. Total reducing power (TRP) assays

Total reducing power estimations were performed based on potassium ferricyanide based reducing assays.^22^ The assay was performed in triplicate, where 200 μL of the test sample (4 mg mL^−1^ DMSO), 400 μL of potassium ferricyanide (1% w/v in H_2_O) and 400 μL of phosphate buffer (0.2 mol L^−1^; pH 6.6) were mixed in an Eppendorf tube. Finally, the reaction mixture was incubated for 20 min at 45 °C. After incubation, 400 μL of trichloroacetic acid (10% w/v in H_2_O) was mixed with the reaction mixture, which was then centrifuged for 10 min at 3000 rpm at room temperature. An aliquot of 150 μL was taken from the supernatant of each mixture and shifted to corresponded well in a 96 micro well plate. Finally, 50 μL 0.1% (w/v) ferricyanide solution was added to each well and absorbance was recorded at 630 nm. Ascorbic acid (40 μL from 1 mg mL^−1^ methanol) and DMSO were taken as a positive control and a blank, respectively. The results were calculated using table curve software (calibration curve) and expressed as micrograms of ascorbic acid equivalent per milligram (AAE μg mg^−1^) of the CeO_2_-NPs.

#### 2.10.4. ABTS assays

The ABTS scavenging activity was evaluated using the ABTS assay (also known as the Trolox antioxidant assay) with minor modifications.^23^ ABTS reaction solution was prepared *via* mixing potassium persulfate (2.45 mM) with 7 mM ABTS salt in equal proportions, followed by incubation for 16 h in the dark. After mixing with test sample, the final reaction mixture was returned to the dark for 15 min at 25 °C. The absorbance values of the test samples were recorded at 734 nm using a microplate reader (BioTek ELX800). Trolox and DMSO were used as positive and negative controls, respectively. The antioxidant potential of a sample was expressed as TEAC, and assays were executed in triplicate.

### 2.11. Biocompatibility with isolated human red blood cells (hRBCs)

The biocompatibility of the CeO_2_-NPs was probed against freshly isolated hRBCs to assess their bio-safe nature.^24^ About 1 mL of blood was collected from healthy individuals with informed consent. The isolated blood was poured into an EDTA tube to prevent blood clotting. For the isolation of erythrocytes, blood was centrifuged at 12 000 rpm for 7 min. Once centrifugation was completed, the supernatant was discarded, and the remaining pellet was washed three times with PBS. 200 μL of erythrocyte suspension was mixed with 9.8 mL of PBS (pH: 7.2) and gently shaken to prepare the PBS–erythrocyte suspension. Different concentrations of NPs along with the prepared erythrocyte suspension were taken in Eppendorf tubes and incubated for 1 h at 35 °C. All the reaction mixtures were centrifuged at 10 000 rpm for 10 min after incubation. 200 μL of supernatant was transferred from each tested sample into a 96-well plate, and the absorption was recorded for haemoglobin release at 540 nm. 0.5% Triton X-100 was used as a positive control, while DMSO was employed as a negative control. Percentage haemolysis was calculated *via*:
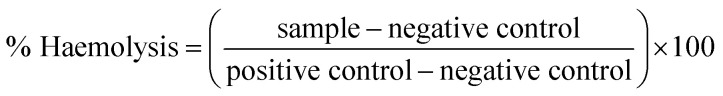
where “sample” and “negative control” represent the absorbance of the sample and negative control, respectively, while “positive control” denotes the absorbance of the positive control.

### 2.12. Live subject statement

The biocompatibility assay of Ag-NPs against human red blood cells includes the use of a human blood sample and was performed in compliance with the relevant laws and institutional guidelines of Bacha Khan University, Pakistan. The ethical committee of Bacha Khan University, Pakistan have approved the experiment and consent form. The blood sample was collected from a healthy volunteer with informed consent.

## 3. Results and discussion

### 3.1. Biosynthesis

**Fig. 1:**
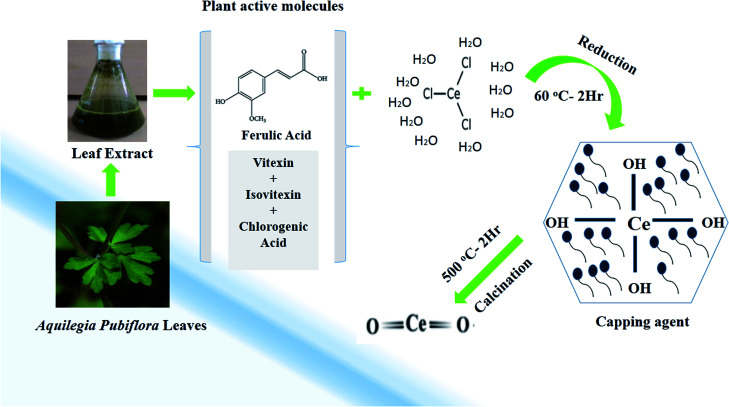
A graphical illustration of the generalized mechanism involved in the *Aquilegia pubiflora* mediated synthesis of the CeO_2_-NPs.

In the study, a simple aqueous leaf extract of *Aquilegia pubiflora* was used as the reducing and stabilizing agent for the preparation of CeO_2_-NPs. To the best of our knowledge, this is the first ever report on the *Aquilegia pubiflora* mediated biosynthesis of nanoceria. The genus *Aquilegia* has more than 60 plant species, which are rich in medicinally important compounds, including berberine, caffeic acid, genkwanin, glochidionolactone-A, ferulic acid, magnoflorine, *p*-coumaric acid and resorcylic acid.^25,26^ These phytochemicals, including phenols and flavonoids, could play an essential role in the synthesis and stability of the CeO_2_-NPs (Fig. 1). The phytochemicals specifically responsible for the reducing and successful capping were identified and quantified using high-performance liquid chromatography (HPLC), as presented in Table 1.

**Table 1:** HPLC analysis results from the biosynthesized CeO_2_-NPs

Compound	mUA min^−1^	DW μg /g^−1^
Vitexin	0.7	3.72
Isovitexin	1.1	8.12
Ferulic acid	0.5	0.143
Chlorogenic acid	4.2	0.129

A total of four phytochemicals, including two flavonoids (vitexin and isovitexin) and two hydroxycinnamic acid derivatives (ferulic acid and chlorogenic acid), were detected and quantified, as shown in Fig. 1 and Table 1. After subsequent washing, drying, grinding and calcination steps, pale yellow CeO_2_-NP powder was obtained, which was stored in an airtight glass vial and labelled as CeO_2_-NPs. The sample was stored at room temperature and further used for physical characterization and investigating its various biological applications.

### 3.2. Physical and morphological characterization

**Fig. 2:**
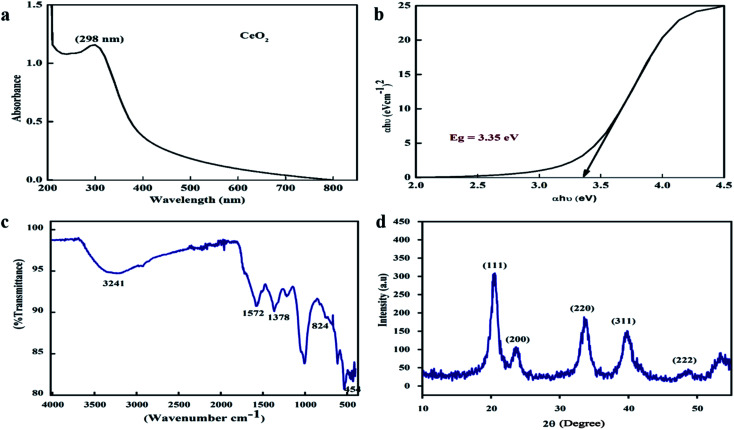
(a) UV-visible spectrum, (b) Tauc plot, (c) FTIR spectra, and (d) XRD pattern of the biosynthesized CeO_2_-NPs.

One of the key characteristics of nanoscale materials is their optical properties. An optical study of CeO_2_ nanoparticles in ethanol was performed using a Schimadzu 1800 UV-vis spectrophotometer. The maximum absorption was observed at 298 nm, as shown in Fig. 2a. The Tauc plot method was used to calculate the band gap, as shown in Fig. 2b, and this was calculated to be 3.35 eV, which is very close to the value reported in the literature.^27^ The band gap depends upon various factors, including the grain size, oxygen deficiency, surface roughness, and lattice strain.^28^

FTIR spectroscopy studies were performed over the range of 400–4000 cm^−1^ to investigate the functional groups and chemical purity of the particles, as shown in Fig. 2c. A broad adsorption band arises in the range of 3241 cm^−1^, which is attributed to the presence of O–H functional groups due to water molecule residue and the formation of Ce(OH)_3_.^29^ The peak at 1572 cm^−1^ indicates the presence of C–O bands due to organic residue within the extract, the peaks at 1634 cm^−1^ and 1582 cm^−1^ arise from scissor bending of O–H groups and C=C bonds, and the peak at 1365 cm^−1^ is due to N–O stretching vibrations.^30,31^ Furthermore, the absorption bands at 824 cm^−1^ and 454.5 cm^−1^ correspond to Ce–O–Ce and Ce–O bond vibrations, respectively, which indicates the successful synthesis of CeO_2_-NPs.^32^

XRD analysis was performed to check the phase purity and crystallinity of the as-synthesized cerium oxide nanoparticles. As shown in Fig. 2d, the sample exhibited four dominant peaks, indexed as (111), (200), (220), and (311), which correspond to the cubic fluorite structure of CeO_2_ (JCPDS no. 89-8436), while a peak of low intensity was also observed as a (222) plane.^33^ No extra peaks were observed in the spectra that could undermine the purity of the nanoscale CeO_2_.

**Fig. 3:**
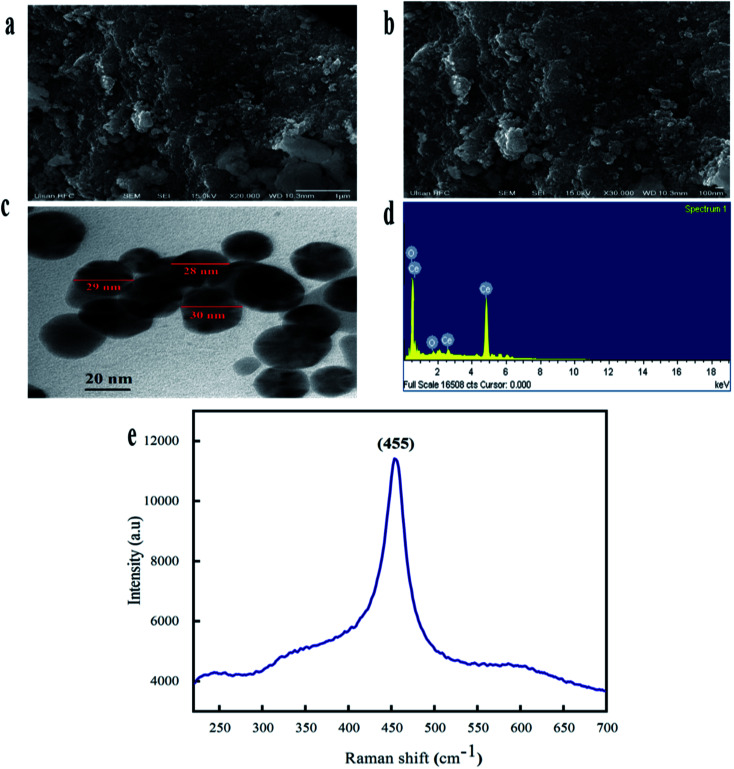
(a) A SEM micrograph with a 1 μm scale bar, (b) a SEM micrograph with a 100 nm scale bar, (c) a TEM micrograph with a 20 nm scale bar, (d) the EDX spectrum, and (e) the Raman spectrum of the biosynthesized CeO_2_-NPs.

The morphological attributes of the greenly synthesized CeO_2_-NPs were examined using scanning electron microscopy (SEM) and transmission electron microscopy (TEM). SEM and TEM micrographs are presented in Fig. 3a and b. The micrographs show that the synthesized nanoparticles exhibit a homogenous phase with spherical morphology.

TEM analysis also confirmed the morphological features of the CeO_2_-NPs. The average particle size, as calculated *via* ImageJ from TEM micrographs, was found to be 28 nm. Similar morphologies of CeO_2_-NPs were also reported in previous studies.^34^

The elemental composition of the CeO_2_-NPs was determined through EDX analysis, as shown in Fig. 3d. The appearance of carbon is attributed to the grid support, while no other significant elements were found in the EDS spectrum apart from Ce and O, affirming the single-phase purity of the CeO_2_-NPs. The as-prepared sample showed weight% values of 73.84% for Ce and 26.16% for O. Raman spectroscopy showed a strong peak at 455 cm^−1^, as depicted in Fig. 3e. This single Raman band is accredited to the presence of a symmetrical stretching mode of Ce–O. The F_2g_ vibration mode of the cubic structure of the CeO_2_-NPs corresponds to this recorded peak.^35^

**Fig. 4:**
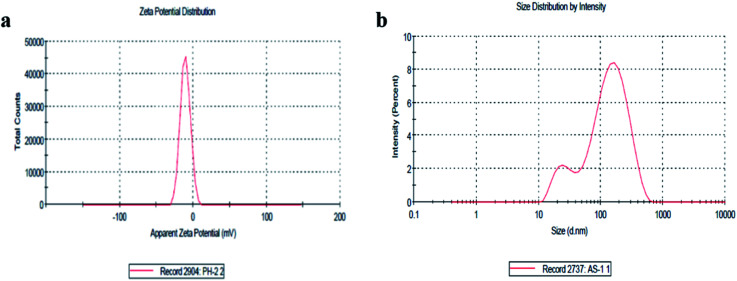
(a) The zeta potential distribution of the CeO_2_-NPs and (b) the size distribution by intensity of the CeO_2_-NPs.

The size distribution and zeta potential (*ζ*) of the biosynthesized CeO_2_-NPs were investigated using the dynamic light scattering (DLS) technique. The zeta potential (*ζ*) defines the colloidal stability and is a typical measurement of the surface charge on a particle. Suspensions that exhibits |*ζ*| ≥ 15 mV are generalized as stable colloids.^36^ In the study, the zeta potential of the CeO_2_-NPs in distilled water (DW) was measured as – 10.5 mV, and this can thus be considered a relatively stable colloidal solution. The zeta potential measurements thus verify and support the dispersion capacity of the greenly synthesized CeO_2_-NPs. The negative surface charge is due to the binding affinity of extract compounds with the NPs, conferring stability on the cerium nanoparticles and alleviating the aggregation potential of the particles.^37^ Moreover, size distribution measurements reveal the average size of the particles to be 86.45 nm. The size distribution graph shows that the particle size is polydispersed and larger compared to that obtained from SEM observations (Fig. 4b). The increased size of the CeO_2_-NPs measured *via* DLS is due to the bias of the technique towards the measurement of larger particles (or even aggregates).^36^

### 3.3. Biological applications

#### 3.3.1. Antibacterial activity

The emergence of ever-increasing antibacterial resistance among many clinically important bacterial species is becoming a major public health concern across the globe. In the United States alone, 2 million cases are identified annually where anti-microbial resistance has placed significant health and economic burdens on both patients and the healthcare system.^38^ The birth of nanotechnology offers promising new tools to combat microbial infectious diseases. In this regard, metal- and metal-oxide-based nanoparticles have shown promising potential for providing novel approaches for antibacterial drug design and combating antibacterial resistance.^39^ CeO_2_-NPs have been studied extensively, recognizing their use as capable antibacterial agents.^40,41^ In the current study, CeO_2_-NP antibacterial assays were performed against five bacterial pathogenic strains, including two Gram positive and three Gram negative bacterial strains, using five different concentrations (5 mg mL^−1^, 4 mg mL^−1^, 2 mg mL^−1^, 1 mg mL^−1^, and 500 μg mL^−1^ of CeO_2_-NPs). Generally, all the bacterial strains were susceptible to the test samples and showed dose dependent inhibition, as illustrated in Table 2. The largest zone of inhibition was measured as 7.11 ± 0.34 mm for *Escherichia coli*, followed by *Staphylococcus epidermidis* (6.81 ± 0.24 mm), and *Klebsiella pneumonia* (6.36 ± 0.29 mm). The smallest zone of inhibition was calculated to be 5.86 ± 0.39 mm for *Bacillus subtilis*. The antibacterial potential of the CeO_2_-NPs is mostly dependent on morphological features, like shape, size and surface area, however, electrostatic interactions between the positively charged NPs and negatively charged bacterial cells play a vital role in determining the bactericidal activity. These electrostatic interactions not only inhibit bacterial growth but they also generate reactive oxygen species (ROS), which lead to cell mortality.

**Table 2:** The antibacterial activities of the CeO_2_-NPs against pathogenic bacterial strains, measured in terms of ZOI (mm)^a^

Pathogenic bacteria	CeO_2_-NPs				
	5 mg mL^−1^	4 mg mL^−1^	2 mg mL^−1^	1 mg mL^−1^	500 μg mL^−1^
*E. coli*	7.11 ± 0.34**	6.81 ± 0.24***	6.36 ± 0.29***	5.86 ± 0.39***	4.17 ± 0.31***
*S. epidermidis*	6.81 ± 0.24***	6.21 ± 0.41***	5.18 ± 0.22***	5.11 ± 0.33***	3.11 ± 0.43***
*K. pneumonia*	6.36 ± 0.29***	5.09 ± 0.39***	5.06 ± 0.31***	4.48 ± 0.28***	3.29 ± 0.41***
*P. aeruginosa*	6.17 ± 0.21***	4.19 ± 0.34***	3.81 ± 0.27***	3.72 ± 0.34***	3.34 ± 0.31***
*B. subtilis*	5.86 ± 0.39***	4.27 ± 0.29***	3.78 ± 0.19***	3.51 ± 0.28***	3.14 ± 0.28***
Positive control (ampicillin)	9.81 ± 0.33	10.11 ± 0.41	8.91 ± 0.34	8.28 ± 0.29	8.31 ± 0.38

a *: highly significant; **: slightly significant; and ***: non-significant difference from the control at *P* < 0.05 *via* one-way ANOVA; values are mean ± SD of triplicate measurements.

#### 3.3.2. Antifungal activity

The antifungal potential of the CeO_2_-NPs was probed against five different spore-forming fungal strains (*Aspergillus niger*, *Aspergillus fumegatus*, *Aspergillus flavus*, *Mucor racemosus*, and *Fusarium solani*) using the well diffusion method. Different concentrations of NP formulations, *i.e.*, 5 mg mL^−1^, 4 mg mL^−1^, 2 mg mL^−1^, 1 mg mL^−1^ and 500 μg mL^−1^, were tested in the experiments. Unlike the bacterial activity, the biogenic CeO_2_-NPs displayed excellent dose-dependent antifungal potential against all strains, as shown in Table 3. Among the strains, *A. niger* was found to be highly susceptible, with a ZOI of 15.1 ± 0.27 mm at 5 mg mL^−1^. At the highest concentration, the zones of inhibition observed for the other strains were 13.9 ± 0.29 mm for *M. racemosus*, 11.9 ± 0.24 mm for *A. fumegatus*, 11.4 ± 0.27 mm for *F. solani*, and 10.1 ± 0.31 mm for *A. flavus*. The exact mechanism of the antifungal activity of the CeO_2_-NPs is not understood, but it is probably due to electromagnetic interactions between the particles and cell surfaces, and ROS generation.^42^ ROS cause the oxidative deterioration of cell membrane lipids,^43^ denaturing the cell membrane permeability with the leakage of potassium ions, ultimately causing cell death.^44^

**Table 3:** The antifungal activities of the CeO_2_-NPs against pathogenic fungi, measured in terms of ZOI (mm)^a^

Pathogenic fungi	CeO_2_-NPs				
	5 mg mL^−1^	4 mg mL^−1^	2 mg mL^−1^	1 mg mL^−1^	500 μg mL^−1^
*A. niger*	15.1 ± 0.27*	11.3 ± 0.33***	7.5 ± 0.16***	4.2 ± 0.11***	3.3 ± 0.12***
*M. racemosus*	13.9 ± 0.29**	8.3 ± 0.31***	5.2 ± 0.23***	3.8 ± 0.17***	3.1 ± 0.14***
*F. solani*	11.4 ± 0.29**	8.1 ± 0.22***	5.7 ± 0.19***	3.7 ± 0.13***	3.1 ± 0.11***
*A. flavus*	10.1 ± 0.31***	7.2 ± 0.19***	6.4 ± 0.11***	4.7 ± 0.16***	4.1 ± 0.15***
*A. fumegatus*	11.9 ± 0.24**	7.4 ± 0.24***	5.7 ± 0.14***	5.1 ± 0.19***	3.2 ± 0.18***
Positive control (amphotericin B)	15.8 ± 0.44	15.4 ± 0.42	14.8 ± 0.38	15.1 ± 0.36	14.8 ± 0.41

a *: highly significant; **: slightly significant; and ***: non-significant difference from the control at *P* < 0.05 *via* one-way ANOVA; values are mean ± SD of triplicate measurements.

#### 3.3.3. Leishmanicidal activity

**Fig. 5:**
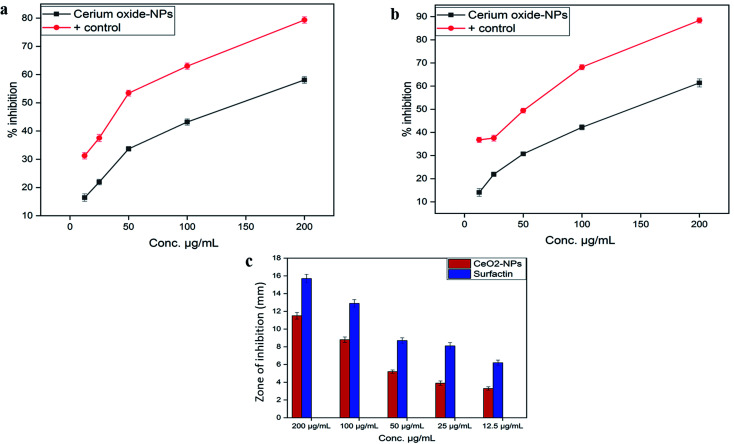
(a) A graphical illustration of the cytotoxic potential of the biosynthesized CeO_2_-NPs against the amastigote form of *Leishmania tropica* (KWH23), (b) a graphical presentation of the cytotoxic potential of the biosynthesized CeO_2_-NPs against the promastigote form of *Leishmania tropica* (KWH23), and (c) a graphical presentation of the protein kinase inhibition activity of *Aquilegia pubiflora* synthesized CeO_2_-NPs.

Leishmaniasis is a neglected, non-contagious, infectious disease caused by parasites, largely found in *Leishmania* species. According to the World Health Organization (WHO), leishmaniasis is one of the six main tropical diseases found in tropical and subtropical regions, with a mortality rate of 50 000 deaths each year.^45^ Due to inappropriate vectors and inefficient and unaffordable drugs, the disease is at high risk of uncontrolled spreading. Recently, metal oxide nanoparticle (oxides of zinc, silver, titanium and magnesium) based treatments have gained popularity because of their significant cytotoxic potential against *Leishmania*.^46^ However, biosynthesized CeO_2_-NPs have been rarely explored for their cytotoxicity against the parasite *Leishmania tropica* (KWH23). In our study, CeO_2_-NP formulations in the concentration range of 12.5–200 μg mL^−1^ were investigated *via* MTT cytotoxic assays against promastigote and amastigote axenic cultures of *Leishmania tropica*. A dose-dependent cytotoxic effect from the CeO_2_-NPs was observed, as presented in Fig. 5a. At the highest concentration of 200 μg mL^−1^, the test sample showed a highest percentage inhibition of 58.11 ± 1.19% against the amastigote form and 61.39 ± 1.73% against the promastigote form of the parasite. The lowest mortality rates observed were 16.43 ± 1.34% and 14.07 ± 1.69% at a tested concentration of 12.5 μg mL^−1^. The IC_50_ values were calculated as 170 μg mL^−1^ for the amastigote form and 130 μg mL^−1^ for the promastigote form. Most studies have shown that metal oxide NPs of smaller size have greater efficacy in inhibiting *Leishmania* by generating more ROS, which induces the process of apoptosis.^47–49^ It has been well documented that *Leishmania* parasites are very sensitive toward ROS, and any agent that has the ability to produce or induce ROS would be considered as a promising leishmanicidal agent.^50^

#### 3.3.4. Kinase inhibition activity

Protein kinases are key enzymes that regulate important cellular processes, including growth and development, as well as cell cycle progression and signal transduction across nuclear membranes.^51,52^ The enzymes are involved in the phosphorylation of serine–threonine and tyrosine residues. These residues are helpful in regulating metabolism, apoptosis, and cellular proliferation/differentiation. Uncontrolled phosphorylation results in genetic abnormalities, leading to tumorigenesis. Protein kinase inhibitors represent a unique class of compounds and are considered as significant anticancer drugs. The biosynthesized CeO_2_-NPs were screened for any protein kinase inhibition activity using the test strain *Streptomyces* 85E. The enzymes are involved in the aerial hyphae formation of the fungus. For preliminary anticancer screening, different concentrations of test samples, *i.e.* from 12.5 μg mL^−1^ to 200 mg mL^−1^, were evaluated for PK inhibition. DMSO and surfactin were used as negative and positive controls, respectively. Generally, dose-dependent inhibition effects were observed (Fig. 5c). The largest zone of inhibition was measured as 11.5 ± 0.37 mm at 200 μg mL^−1^, while the smallest inhibition zone, *i.e.* 3.3 ± 0.19 mm, was achieved at 12.5 μg mL^−1^. The considerable kinase inhibition potential led us to further investigate the CeO_2_-NPs for *in vitro* anticancer activity.

#### 3.3.5. Anti-proliferative effects against the HepG2 cell line

**Fig. 6:**
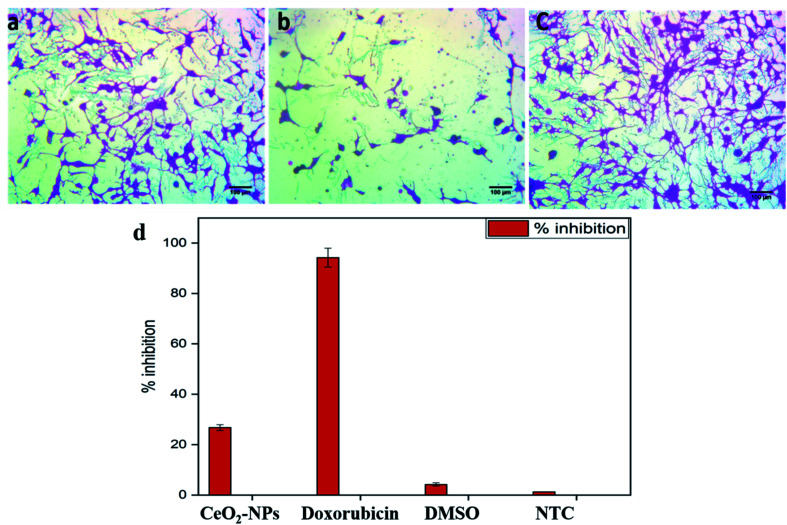
Microscopic images of HepG2 cells showing (a) CeO_2_-NP-treated cells, (b) DOX-treated cells, and (c) NTC. Cells were treated with 100 μg mL^−1^ NPs for 24 h; untreated cells and doxorubicin (30 μM) were included as controls; magnification = 200×; scale bar = 100 μm. (d) A graphical representation of the cytotoxic potential of the biosynthesized CeO_2_-NPs against the HepG2 cell line at 100 μg mL^−1^.

Hepatocellular carcinoma (HCC), the most commonly diagnosed cancer, is the second leading cause of cancer-related deaths, with 7 million deaths per year.^52^ CeO_2_-NPs are associated with anticancer effects *via* inducing oxidative stress and apoptosis in cancer cells while protecting normal cells.^53^ It is suggested that CeO_2_-NPs support reactive oxygen species (ROS) production in cancerous cells (at acidic pH) by converting from a +3 oxidation state to a +4 oxidation state, while scavenging ROS in normal cells *via* reconversion from a +4 oxidation state to a +3 oxidation state.^54^ In addition, anti-angiogenic and anti-invasive properties have also been reported. Such characteristics make CeO_2_-NPs a novel attractive tool for cancer therapeutics. Accordingly, in this study, the biosynthesized CeO_2_-NPs were evaluated for their anticancer effects on the human HepG2 cell line using MTT assays at a screening concentration of 100 μg mL^−1^. To the best of our knowledge, this is the first ever report on the *in vitro* anti-hepatocarcinoma effects of greenly synthesized nanoceria. The anti-proliferative effects of the CeO_2_-NPs were shown to effectively reduce the viability of the HepG2 cell line (Fig. 6), with inhibition of 26.78% ± 1.16%. Compared with the CeO_2_-NPs, the positive control doxorubicin and the negative control DMSO resulted in 94.24% ± 3.75% and 4.24% ± 0.63% inhibition, respectively. Our findings thus suggest that the biosynthesized CeO_2_-NPs could be used as a novel therapeutic agent, either in pristine form or as vehicles against hepatocarcinoma. However, detailed *in vitro* and *in vivo* studies should be designed to augment the results and to investigate the mechanism of the anticancer effects.

#### 3.3.6. *In vitro* antidiabetic studies

Diabetes mellitus (DM) is a metabolic disorder that is mainly characterized by chronic hyperglycemia, which is either due to reduced insulin production or the insensitivity of cells towards available insulin. According to a recent report from the International Diabetes Federation (IDF), 425 million adults are living with diabetes mellitus and the number could rise to 629 million by 2045.^55^ The inhibition of two important carbohydrate-hydrolyzing enzymes in the digestive tract, *i.e.* alpha amylase and alpha glucosidase, offers an effective therapeutic approach to lowering postprandial hyperglycemia.^56^ In the study, different concentrations of biosynthesized CeO_2_-NPs ranging from 200–12.5 μg mL^−1^ were tested for α-amylase and α-glucosidase inhibition. Our findings (Table 4) indicate that moderate/low α-amylase and α-glucosidase inhibition activity arises when using the biosynthesized NPs. Maximum inhibition of about 39% ± 0.24% was observed at the highest concentration of 200 μg mL^−1^ for α-amylase, while 31.28% ± 0.49% inhibition was observed for α-glucosidase. The % inhibition decreased at subsequent lower concentrations.

**Table 4:** The α-amylase and α-glucosidase inhibitory potentials (%) of the CeO_2_-NPs

Conc. (μg mL^−1^)	α-Amylase		α-Glucosidase	
	CeO_2_-NPs	Acarbose	CeO_2_-NPs	Acarbose
200	39 ± 0.24	89.40 ± 1.24	31.2 ± 0.49	84.34 ± 1.92
100	27.3 ± 0.31	73.20 ± 1.51	21.6 ± 0.33	65.98 ± 1.67
50	21.1 ± 0.19	59.43 ± 1.09	13.9 ± 0.24	53.45 ± 1.11
25	12.4 ± 0.17	44.87 ± 0.93	7.2 ± 0.16	23.4 ± 1.27
12.5	5.9 ± 0.11	27.78 ± 0.66	0	17.32 ± 0.73

#### 3.3.7. Antioxidant potential

Total antioxidant capacity (TAC), total reducing power (TRP), and DPPH-free radical scavenging and 2,2′-azino-bis(3-ethylbenzothiazoline-6-sulphonic acid) ABTS assays were performed to screen the *in vitro* antioxidant potentials of the biosynthesized CeO_2_-NPs at concentrations ranging from 12.5–200 μg mL^−1^ (Table 5). In general, the NPs displayed concentration-dependent antioxidant potentials during all the performed assays. The total antioxidant capacity (TAC) is based on ROS species.^57^ The maximum value for total antioxidants, in terms of ascorbic acid equivalents of the tested sample, was found to be 52.71 ± 1.14 μg AAE per mg at the highest concentration used. To further assess the antioxidant capacity, total reducing power estimation (TRP) assays were performed. A substance possessing redox properties neutralizes and absorbs free radicals *via* the transformation of Fe^3+^ ions to Fe^2+^ ions. Thus, NPs exhibiting reducing power have the capacity to reduce ferric ions into ferrous ions.^58^ The highest reducing power potential observed was 87.06 ± 1.24 AAE per mg. Subsequently, DPPH (2,2-diphenyl-1-picrylhydrazyl) free radical scavenging and ABTS (2,2′-azino-bis(3-ethylbenzothiazoline-6-sulphonic acid)) assays were performed to augment the TAC and TRP results. The spectrophotometric methods used are based on the quenching of stable coloured radicals of DPPH and ABTS, indicating the antioxidant scavenging abilities.^59^ Similarly, the highest DPPH and ABTS scavenging activities were observed at 200 μg mL^−1^, *i.e.* 38% ± 1.38%, and 74.21% ± 2.64% TEAC (Trolox equivalent antioxidant capacity), respectively. From the summarized results, it can be suggested that the biosynthesized CeO_2_-NPs exhibit considerable *in vitro* antioxidant abilities.

**Table 5:** The antioxidant potential of the synthesized *A. pubiflora*-based CeO_2_-NPs

Conc. (μg mL^−1^)	TAC (μg AAE per mg)	TRP (μg AAE per mg)	ABTS (TEAC)	DPPH (% FRSA)
200	52.71 ± 1.14	87.06 ± 1.24	74.21 ± 2.64	38.72 ± 1.38
100	34.64 ± 1.32	59.05 ± 0.98	63.31 ± 1.99	24.33 ± 1.2
50	26.39 ± 0.84	52.43 ± 0.92	54.48 ± 2.25	21.18 ± 1.74
25	21.51 ± 0.71	31.87 ± 0.95	39.71 ± 2.21	12.32 ± 0.97
12.5	16.93 ± 1.22	27.64 ± 1.13	21.90 ± 1.91	9.61 ± 1.82

#### 3.3.8. Biocompatibility

To investigate their bio-safe and biocompatible nature, the greenly synthesized CeO_2_-NPs were screened for biocompatibility against human red blood cells (hRBCs). In the experiments, freshly isolated human red blood cells (hRBCs) and NP formulations of different concentrations (25–400 μg mL^−1^) were co-incubated in phosphate buffer saline (PBS), which mimics the extracellular environment.^60^ The assay is based on the release and measurement of hemoglobin from red blood cells (RBCs), which can be prompted by NPs if the particles have the ability to rupture the RBCs.^61^ In principle, hemolysis ≥ 5% is considered hemolytic while hemolysis ≤ 5% is considered non-hemolytic. In the study, the tested NPs exhibited remarkable hemocompatibility, even at the highest concentration used (Table 6). The study thus concludes that the biosynthesized CeO_2_-NPs are highly biocompatible and do not induce toxic effects against isolated hRBCs. However, detailed *in vivo* studies must be designed to verify and augment these *in vitro* results.

**Table 6:** % Hemolysis induced by the synthesized *A. pubiflora*-based CeO_2_-NPs

S. no.	Concentration (μg mL^−1^)	% Hemolysis
1	400	1.74 ± 0.12
2	200	1.15 ± 0.18
3	100	0.62 ± 0.20
4	50	0.28 ± 0.20

## 4. Conclusions

In the study, we have demonstrated, for the first time, the use of the medicinally important plant *Aquilegia pubiflora* in the synthesis of highly biocompatible CeO_2_-NPs with diverse biomedical applications. A green chemistry approach was followed, which is highly facile, economical and ecofriendly. The synthesized particles exhibited high stability, purity, and spherical morphology, with an average size of 28 nm. The nanoparticles had average bactericidal properties but exhibited excellent antifungal potential, especially against *Aspergillus niger* and *Mucor racemosus*. The particles were also found to be highly active against both the amastigote (IC_50_: 114 μg mL^−1^) and promastigote (IC_50_: 97 μg mL^−1^) forms of the leishmanial parasite *Leishmania tropica* (KWH23). Moreover, considerable anticancer potential was shown against hepatocarcinoma (the HepG2 cell line). Hemocompatibility studies revealed that the NPs were highly compatible with human red blood cells. Our study thus concludes that the synthesized *Aquilegia pubiflora* based CeO_2_-NPs are highly biocompatible and show promising potential for use in alternative leishmaniasis and cancer therapeutics. Moreover, their considerable antimicrobial and antioxidant potential mean these CeO_2_-NPs could be novel nanotools for diverse biomedical applications.

## Ethical approval

This article does not contain any direct studies on human participants and animals. The experiment was performed in compliance with the relevant laws and institutional guidelines of Bacha Khan University, Charsadda with prior approval from the ethical committee. Only the RBC haemolysis assay includes the use of a human blood sample and was performed with donor consent and according to institutional guidelines.

## Conflicts of interest

The authors declare no competing interests.

## Supplementary Material
